# Thermal Behaviors and Interaction Mechanism of Ammonium Dinitramide with Nitrocellulose

**DOI:** 10.3390/molecules28052346

**Published:** 2023-03-03

**Authors:** Qiong Wang, Xiao-Hong Wang, Qing Pan, Hai Chang, Hong-Jian Yu, Wei-Qiang Pang

**Affiliations:** Xi’an Modern Chemistry Research Institute, Xi’an 710065, China

**Keywords:** ammonium dinitramide, nitrocellulose, interaction, thermal decomposition

## Abstract

The initial interaction mechanism is very important for the design and safety of nano-scale composite energetic materials composed of ammonium dinitramide (ADN) and nitrocellulose (NC). The thermal behaviors of ADN, NC and an NC/ADN mixture under different conditions were studied by using differential scanning calorimetry (DSC) with sealed crucibles, an accelerating rate calorimeter (ARC), a self-developed gas pressure measurement instrument and a DSC-thermogravimetry (TG)—quadrupole mass spectroscopy (MS)—Fourier transform infrared spectroscopy (FTIR) combined technique. The results show that the exothermic peak temperature of the NC/ADN mixture shifted forward greatly in both open and closed circumstances compared to those of NC or ADN. After 585.5 min under quasi-adiabatic conditions, the NC/ADN mixture stepped into the self-heating stage at 106.4 °C, which was much less than the initial temperatures of NC or ADN. The significant reduction in net pressure increment of NC, ADN and the NC/ADN mixture under vacuum indicates that ADN initiated the interaction of NC with ADN. Compared to gas products of NC or ADN, two new kinds of oxidative gases O_2_ and HNO_2_ appeared for the NC/ADN mixture, while NH_3_ and aldehyde disappeared. The mixing of NC with ADN did not change the initial decomposition pathway of either, but NC made ADN more inclined to decompose into N_2_O, which resulted in the formation of oxidative gases O_2_ and HNO_2_. The thermal decomposition of ADN dominated the initial thermal decomposition stage of the NC/ADN mixture, followed by the oxidation of NC and the cation of ADN.

## 1. Introduction

Nitrocellulose (NC), as a classical energetic macromolecule with high enthalpy of formation (−2767 J/g for NC with 11.97 N%), is widely used as one of the most important ingredients for double-base solid propellant [1,2] and gun propellant [3,4]. The higher reactivity of nanosized NC has great potential to improve the combustion performance of propellants or explosives [5,6]. In addition to that, as common NC fibers are downscaled to nanosized particles, the friction sensitivity can be improved substantially [7]. As a brand new oxidizer, ammonium dinitramide (ADN) with oxygen balance 25.80% [8] was attempted to replace ammonium perchlorate (AP) in propellant [9,10], and it was found that the addition of ADN can increase the specific impulse and burning rate of propellants. Apparently, the combination of highly energetic materials NC and ADN has great potential to improve the energy characteristics and combustion behaviors of solid propellants due to the higher reactivity between fuel and oxidizer. The latest research [11] shows that as ADN mixes with NC at the micro/nano-scale level, ADN can improve the combustion performance of micro/nano-scale NC fibers significantly and maintain the sensitivity level of NC itself. However, investigation on the compatibility of ADN with NC via differential scanning calorimetry (DSC) and vacuum stability testing found that ADN interacted strongly with NC [12,13,14,15], which prevented the further application of ADN in solid propellant together with NC. There are many investigations on the thermal behavior and thermal decomposition mechanisms of NC [16,17,18,19,20] or ADN [21,22,23,24,25,26,27] alone, while, the interaction mechanism between NC and ADN has not been reported as of yet. As the chemical compatibility arising from the chemical reactions between ADN and NC is very important for the manufacturing safety and storage safety of composite nano-energetic materials; hence, it is necessary to excavate the root cause that leads to the incompatibility of ADN with NC in order to lay the foundation for improving the compatibility of NC with ADN by NC modification.

As is known to all, the initiation reaction is critical to screening stabilizers when taking precautions to inhibit the initiation reaction or modifying molecular properties to improve the compatibility of NC with ADN. In this paper, the thermal behaviors of ADN, NC and the NC/ADN mixture under different conditions were investigated using DSC, gas pressure measurement (GPM) and an accelerating rate calorimeter (ARC). More importantly, we attempted to discern the initial interaction mechanism between ADN and NC from variations in gas products by using the combined technology of DSC-thermogravimetry (TG) and quadrupole mass spectroscopy (MS) coupled with Fourier transform infrared spectroscopy (FTIR).

## 2. Results and Discussion

### 2.1. Thermal Behavior in Open Circumstances

**Figure 1:**
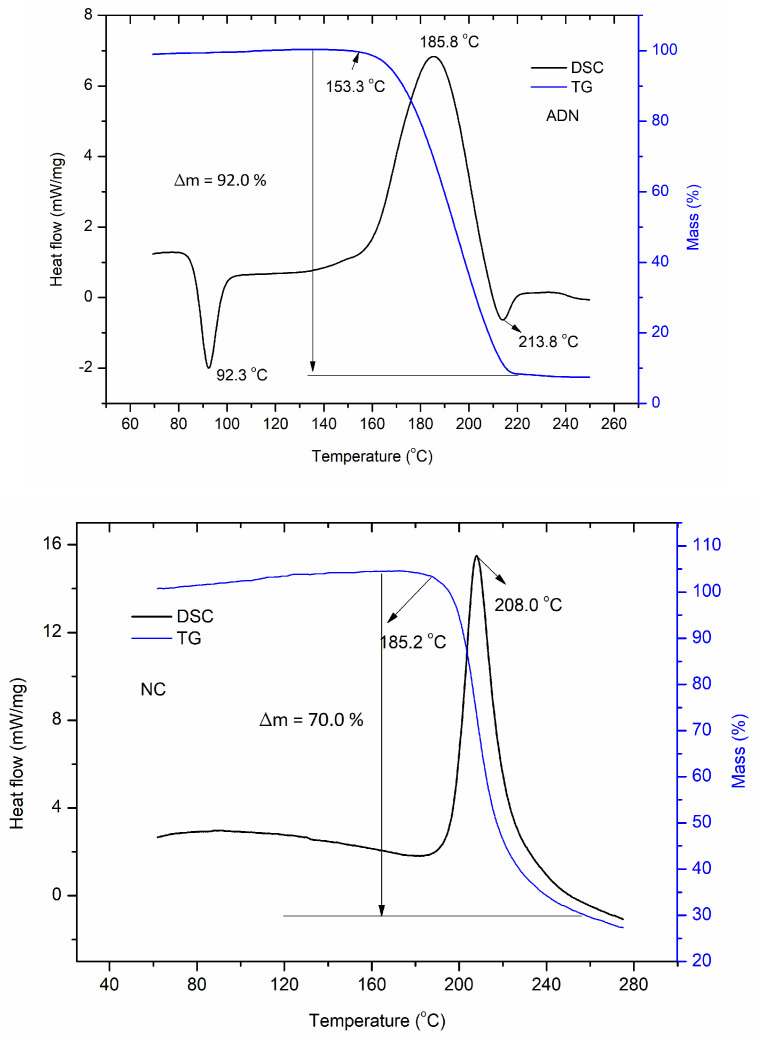

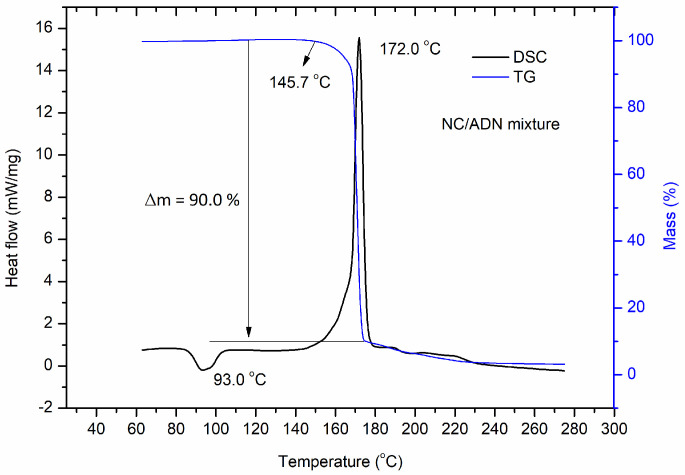
DSC-TG curves of ADN, NC and NC/ADN mixture in open circumstances.

The DSC-TG curves of ADN, NC and the NC/ADN mixture are shown in Figure 1.

It can be seen from Figure 1 that ADN melted at 92.3 °C and then stepped into a slow exothermic thermal decomposition process until 153.3 °C, which was followed by a rapid exothermic process with the peak temperature at 185.8 °C. After the main exothermic thermal decomposition process, a minor endothermic thermal decomposition process appeared with the peak temperature at 213.8 °C, which can be ascribed to the evaporation of thermal decomposition of product AN [28,29] formed in the heating process of ADN [30]. The mass loss in the main thermal decomposition stage of ADN reached 92.0%. NC decomposed in one step with the exothermic peak temperature at 208.0 °C and a mass loss of 70.0%. For the NC/ADN mixture, it melted at 93.0 °C and decomposed mainly in one step with an exothermic peak temperature at 172.0 °C and a mass loss of 90.0%, which indicates that NC decomposed more completely when mixed with ADN. The exothermic peak temperature of the NC/ADN mixture (172.0 °C) was 36.0 °C less than that of NC and 13.8 °C less than that of ADN. The fast mass-loss process of the NC/ADN mixture began at 145.7 °C, which was 39.5 °C less than that of NC and 7.6 °C less than that of ADN. The significant differences of the NC/ADN mixture with NC or ADN in terms of mass loss, the initial temperature of fast mass loss and the exothermic peak temperature indicate that strong interactions happened between NC and ADN. 

### 2.2. Thermal Behavior in Close Circumstances

**Figure 2:**
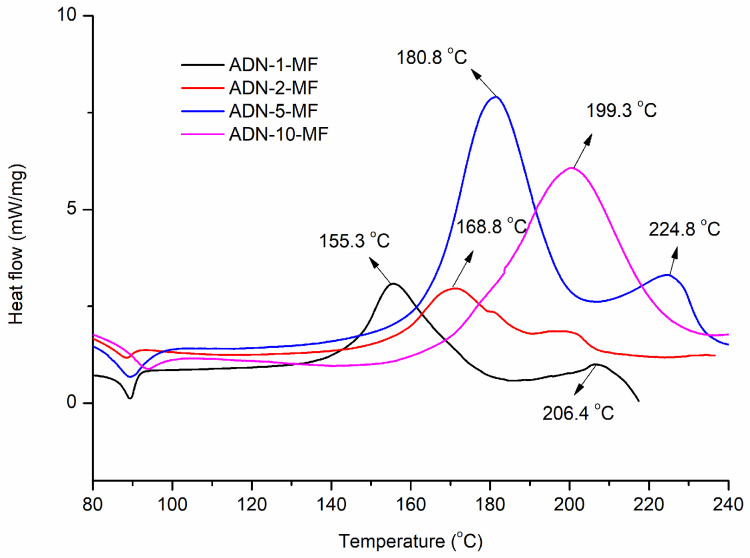

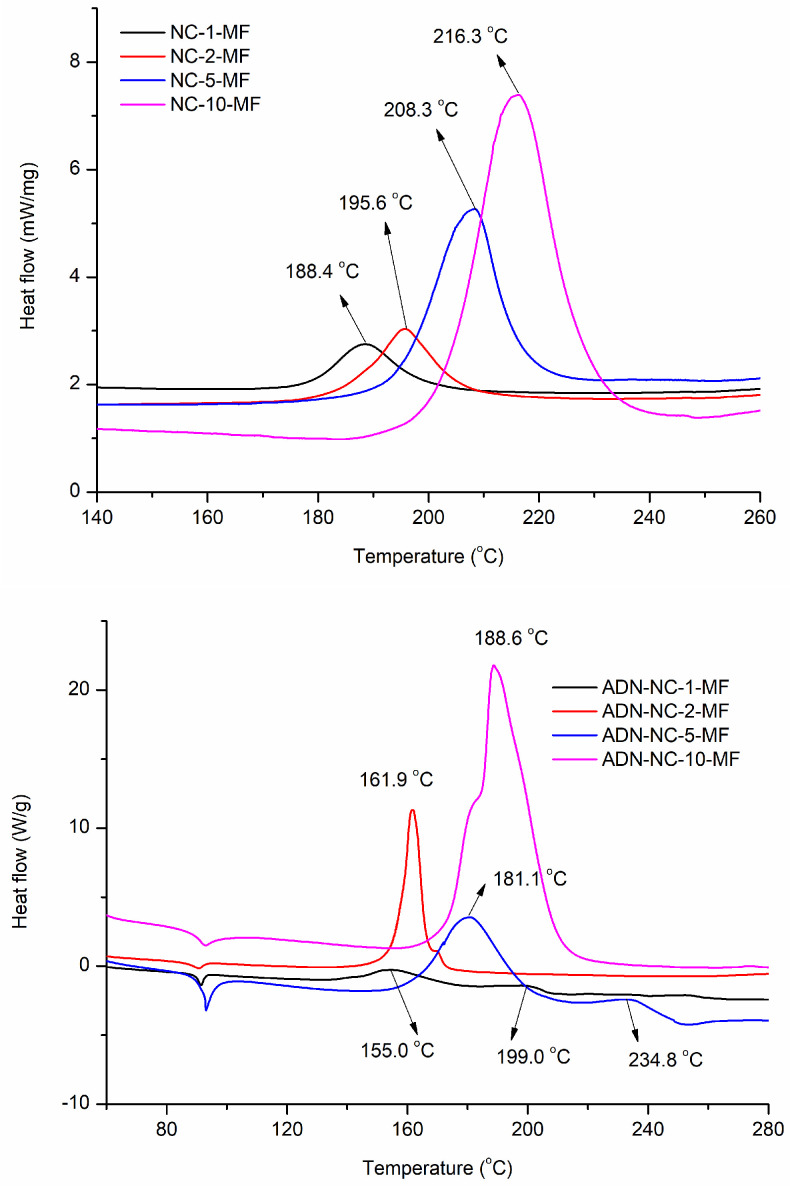
DSC curves of ADN, NC and NC/ADN mixture in closed circumstances.

The DSC tests of ADN, NC and the NC/ADN mixture using hermetic crucibles at different heating rates were explored, and the curves are shown in Figure 2. The apparent activation energy (*E*_a_) was calculated by using the Kissinger method [31], and the results were listed in Table 1.

It can be seen from Figure 2 that the DSC exothermic peak temperatures of ADN, NC and the NC/ADN mixture in a closed crucible at a heating rate of 10 °C/min moved back compared to those in an open crucible. This phenomenon can be ascribed to the exothermic reactions between the confined gases in the sealed crucible. The same as in the open circumstance, the DSC peak temperature of the NC/ADN mixture in closed circumstances at a heating rate of 10 °C/min was still the lowest compared to those of ADN or NC. A secondary exothermic process appeared, which was recognized as the thermal decomposition of AN, an important thermal decomposition product of ADN. Correspondingly, a small exothermic process behind the main exothermic process also existed on the DSC curves of the NC/ADN mixture. The *E*_a_ of the NC/ADN mixture was 96.8 kJ/mol, which was higher than that of ADN (83.2 kJ/mol) and lower than that of NC (143.7 kJ/mol).

### 2.3. Thermal Behavior in Quasi-Adiabatic Circumstances

**Figure 3:**
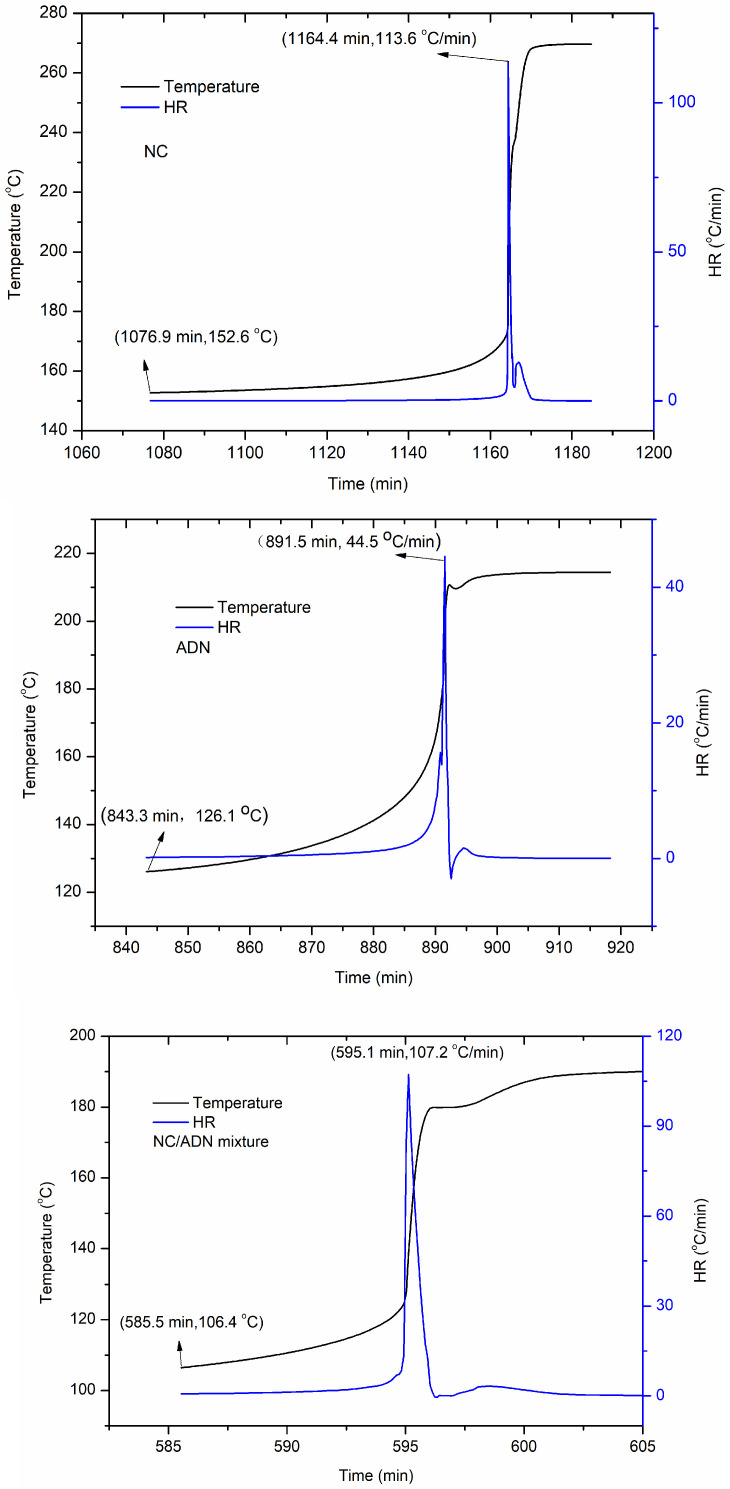
Thermal decomposition curves of ADN, NC and NC/ADN mixture under quasi-adiabatic circumstances.

The thermal behavior of ADN, NC and the NC/ADN mixture in quasi-adiabatic circumstances were conducted. The results are shown in Figure 3, and the characteristic parameters are listed in Table 2.

It can be seen from Figure 3 and Table 2 that the heating rate at the initial detection point of the NC/ADN mixture reached 0.70 °C/min after being heated for 585.5 min. The temperature at the initial detection point of the NC/ADN mixture was 106.4 °C, which was 19.7 °C less than that of ADN and 46.2 °C less than that of NC. The temperature rate of the NC/ADN mixture reached its maximum after heating for another 9.6 min; however, the times were 48.2 min and 87.5 min for ADN and NC, respectively, which indicates that the NC/ADN mixture was easier to decompose compared to ADN or NC under quasi-adiabatic conditions.

### 2.4. Thermal Behavior under Constant Temperature Conditions

**Figure 4:**
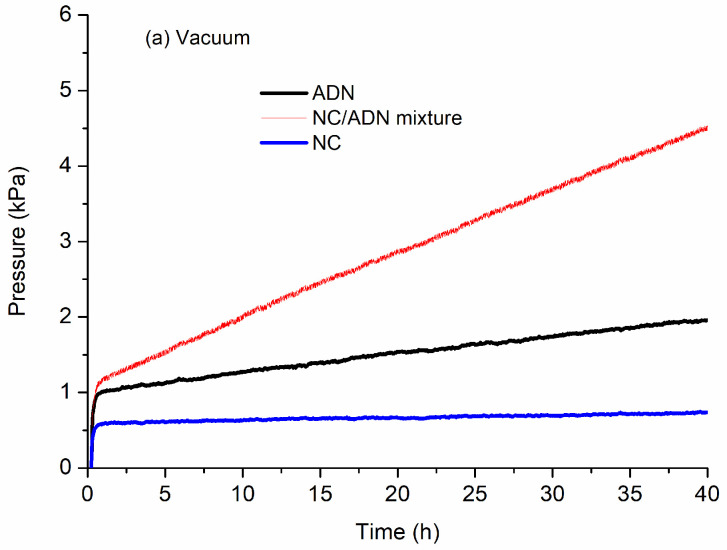

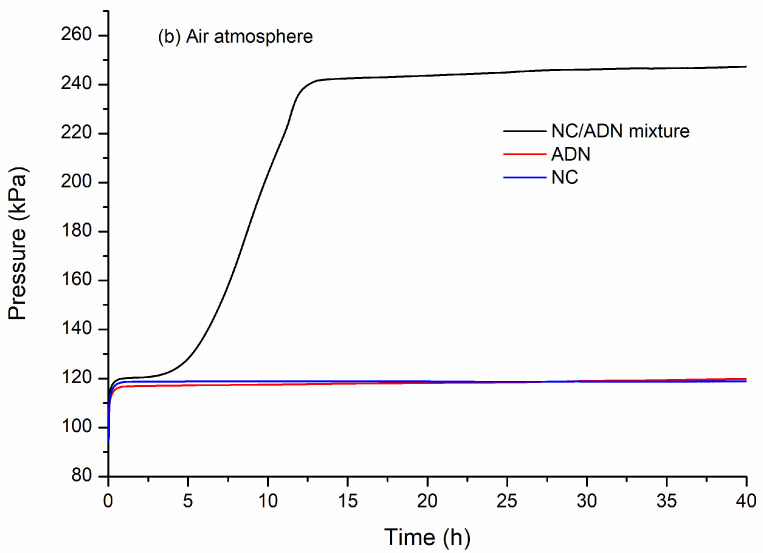
The thermal decomposition gas pressure vs. time curves of ADN, NC and NC/ADN mixture.

The thermal decomposition gas pressures of ADN, NC and the NC/ADN mixture under constant temperature conditions with two initial states, i.e., vacuum and air atmosphere, were measured. The pressure vs. time curves are shown in Figure 4, and the characteristic pressures are listed in Table 3.

The difference in the NC/ADN mixture pressure curves under vacuum and air can be seen from Figure 4. The pressure increase of the NC/ADN mixture after 40 h under vacuum conditions was only about 3.39 kPa, while the pressure increase of the NC/ADN mixture under air conditions was 127.16 kPa, which indicates that the initial pressure had great effects on the thermal decomposition of the NC/ADN mixture. The significant pressure increase in the NC/ADN mixture under air atmosphere indicates that NC interacted intensively with ADN. However, intensive interactions were retarded under high vacuum conditions. Comparing the pressure increases of NC under vacuum and air atmosphere, it can be seen that the initial state hardly had effect on the thermal decomposition of NC. However, for ADN, under vacuum, the net pressure increment was 0.95 kPa, while in contrast, the pressure increase under air atmosphere was 2.91 kPa, which indicates that the initial pressure also had great influence on the decomposition of ADN. Simultaneously, it can be concluded that ADN decomposition, especially the primary products of ADN, had critical effects on the interaction of ADN with NC.

### 2.5. Mass Spectroscopy Detection of Thermal Decomposition Gas Products

The thermal decomposition product fragments of ADN, NC and the NC/ADN mixture were detected using MS, and the results are listed in Table 4, Table 5 and Table 6.

It can be seen from Table 4 that the thermal decomposition products of ADN were mainly comprised of H_2_O, N_2_, N_2_O, NH_3_ and NO with highest intensities of 21.4 × 10^11^, 21.1 × 10^11^, 7.22 × 10^11^, 4.00 × 10^11^ and 2.74 × 10^11^, respectively. As shown in Table 5, the thermal decomposition products of NC mainly comprised N_2_/CO, H_2_O, N_2_O/CO_2_, NO/CH_2_O and HCN with highest intensities of 26.5 × 10^11^, 19.3 × 10^11^, 6.35 × 10^11^, 4.87 × 10^11^ and 0.65 × 10^11^, respectively. For the NC/ADN mixture, as shown in Table 6, the thermal decomposition products mainly contained N_2_O/CO_2_, H_2_O, N_2_/CO, OH, NO, O_2_ and NO_2_ with highest intensities of 239 × 10^11^, 61.0 × 10^11^, 56.0 × 10^11^, 34.5 × 10^11^, 19.3 × 10^11^, 7.69 × 10^11^ and 1.77 × 10^11^, respectively. Different from those of ADN or NC, the fragments of HNO_2_ and O_2_, detected for the first time, appeared as the main decomposition products of the NC/ADN mixture.

On the whole, all the initial temperatures and peak temperatures of the NC/ADN mixture were brought forward compared to those of ADN or NC. The NO_2_ formation of the NC/ADN mixture began at 144.8 °C, which was 3.1 °C less and 25.7 °C less than in ADN and NC, respectively. The initial temperature of N_2_O formation of ADN (146.9 °C) approximated that of the NC/ADN mixture (147.8 °C), which indicates that the thermal decomposition of ADN dominated the first thermal decomposition process of the NC/ADN mixture. Compared to ADN, the highest intensities of fragments N_2_O/CO_2_, NO_2_, H_2_O and N_2_/CO in the NC/ADN mixture increased by 33.1, 25.3, 2.85 and 2.65 times, respectively. Instead of N_2_/CO and H_2_O, the fragment N_2_O/CO_2_ became the main product with the highest intensity, almost 135.0 times the level of NO_2_, which indicates that the thermal decomposition of ADN through the rearrangement anion to form N_2_O was much preferred as ADN mixed with NC [26].

### 2.6. FTIR Spectroscopy Detection of Thermal Decomposition Gas Products

**Figure 5:**
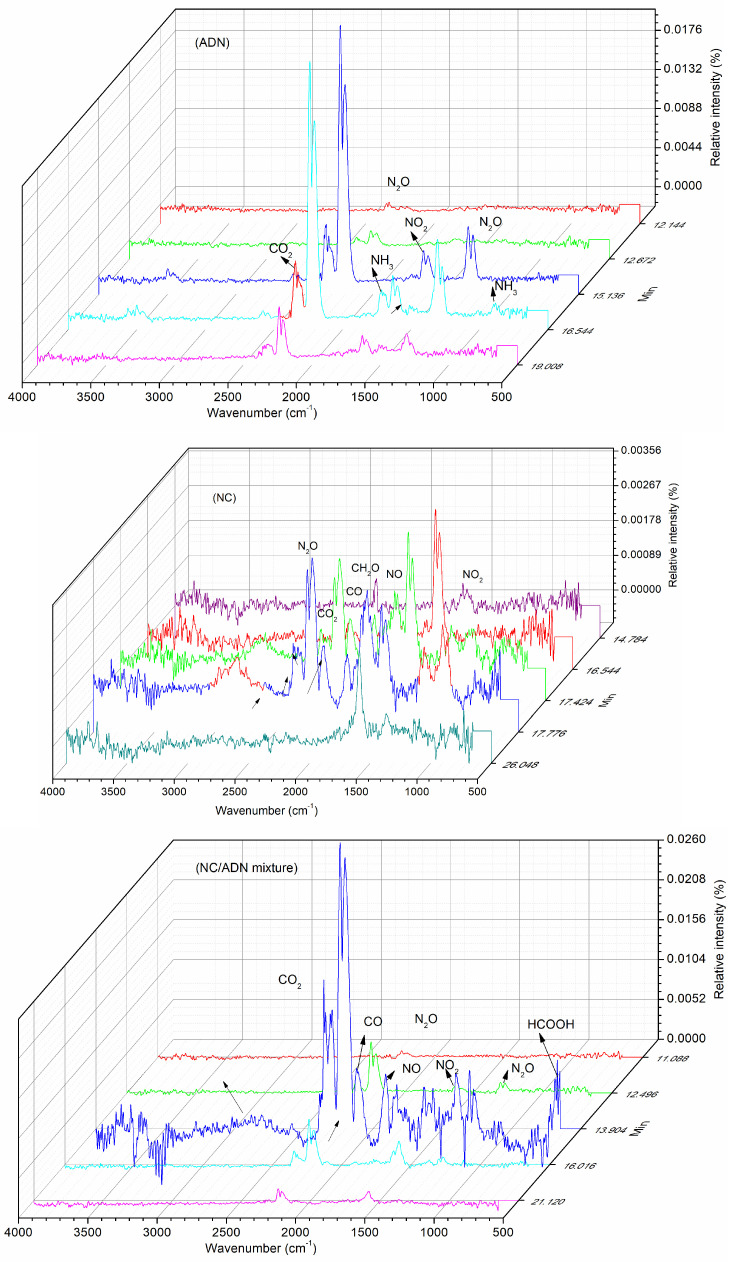
Gas products IR spectroscopy of ADN, NC and NC/ADN mixture at different moments.

**Figure 6:**
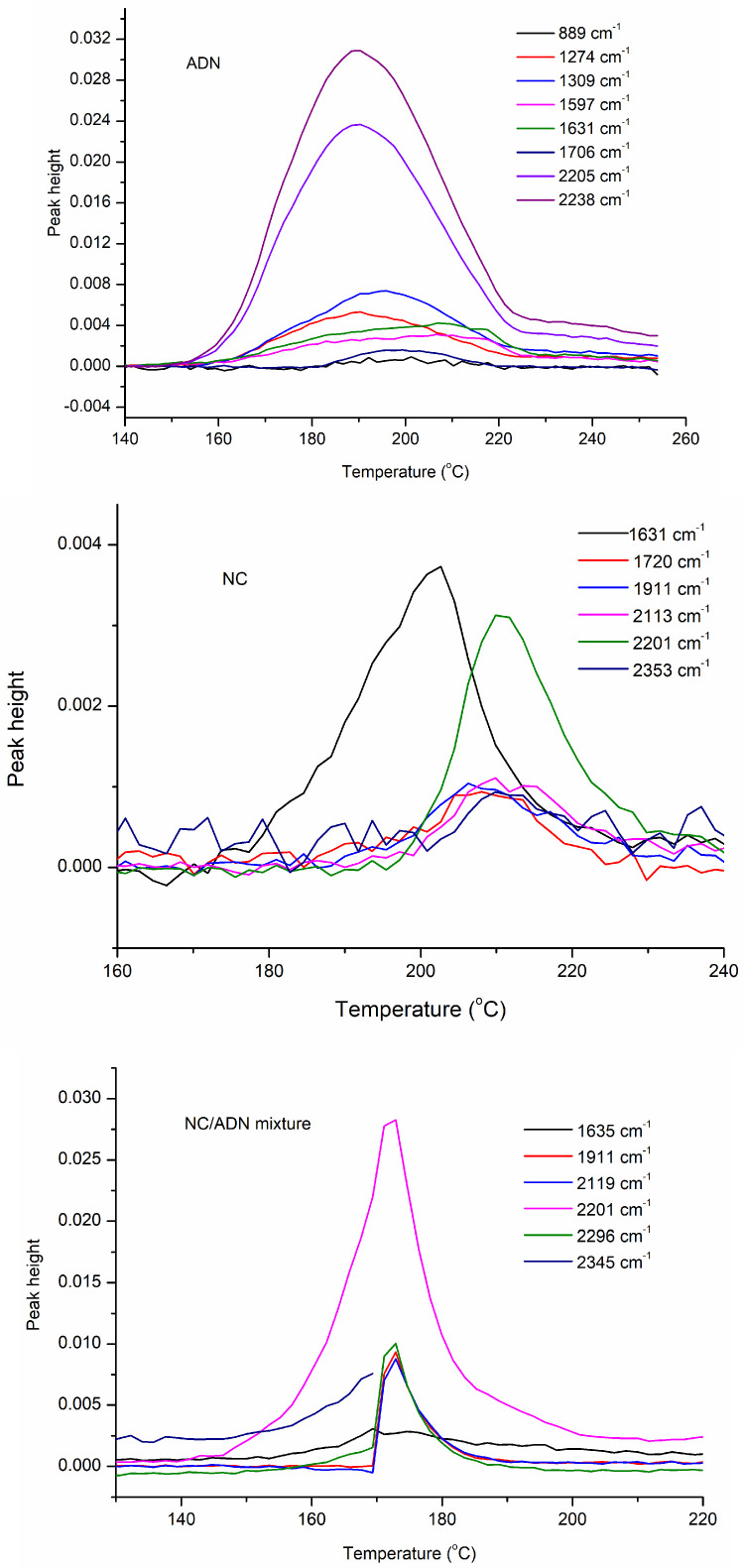

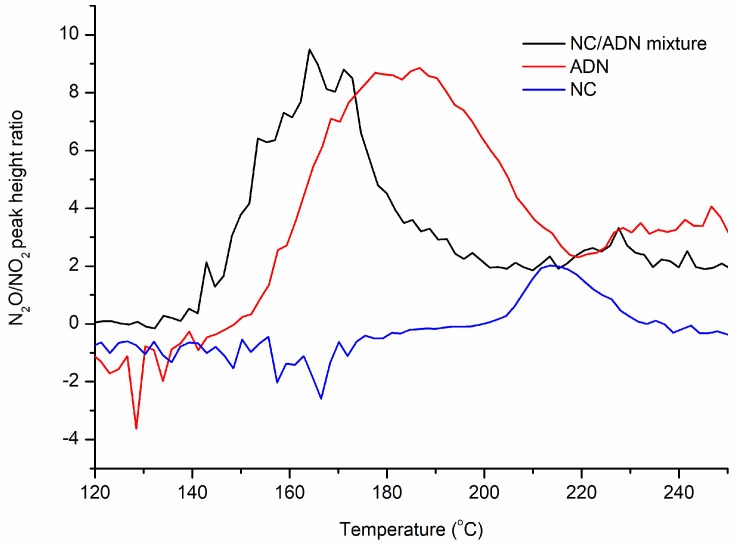
The IR absorption peak height vs. temperature of the main gas products of ADN, NC and NC/ADN mixture.

The thermal decomposition gas products of ADN, NC and the NC/ADN mixture were detected via infrared spectrometer. The characteristic absorption wavelengths and the assignments are listed in Table 7. The IR spectroscopy of gas products at different moments are shown in Figure 5, and the IR absorbance peak heights vs. temperature are shown in Figure 6.

It can be seen that the main IR-active thermal decomposition products of ADN included NH_3_, N_2_O and NO_2_, wherein the N_2_O intensity was the highest. It was found that NO_2_ formed firstly at 141.1 °C (1631 cm^−1^) through the break of the N-NO_2_ bond, then N_2_O began to form at 144.8 °C (1274 cm^−1^) by the combination of the central nitrogen atom and the nitrogen on the NO_2_ group through ADN anion rearrangement [26]. Until the temperature reached 177.6 °C, NH_3_ (889 cm^−1^) appeared. As NH_3_ can become volatile quickly once formed, we speculate that ADN decomposed in the form of ion NH_4_^+^N(NO_2_)_2_^−^ without proton transfer from the cation to anion to form H^+^N(NO_2_)_2_^−^ (HDN) and NH_3_ from 141.1 °C to 177.6 °C after the ADN melted. Due to the ADN molecule not containing the carbon element, the appearance of CO_2_ (2343 cm^−1^) at 142.1 °C can be ascribed to the oxidation of the processing aid, which was introduced to assist ADN crystallization.

The same as described in the literature [16,17,32], the main thermal decomposition gas products of NC comprised NO_2_, N_2_O, NO, CO, CO_2_ and aldehyde (CH_2_O) or carboxylic acid (HCOOH), wherein the intensity of NO_2_ and N_2_O were the highest. NC started to decompose with the dissociation of the O-NO_2_ bond and formed NO_2_ gas, which was detected via the IR detector at 166.5 °C (1631 cm^−1^). The carbon–oxygen skeleton of the cellulose was oxidized by NO_2_ to form aldehyde or carboxylic acid, which can be found at 184.6 °C (1720 cm^−1^) followed by the formation of NO at 186.4 °C (1911 cm^−1^) and CO at 189.9 °C (2113 cm^−1^). Further reaction of the formed intermediate products produced the N_2_O at 195.4 °C (2201 cm^−1^), CO_2_ at 200.8 °C (2353 cm^−1^) and HCN at 202.7 °C (714 cm^−1^).

Furthermore, the main IR-active gases of the NC/ADN mixture included N_2_O, NO_2_, NO, CO and CO_2_. The absorbance (1390 cm^−1^) appearing at 13.904 min can probably be ascribed to the formation of HNO_2_ or HNO_3_. However, the signal of NH_3_ released from ADN and aldehyde or carboxylic acid produced by NC thermal decomposition became too weak to be detected. It can be speculated from the IR result that the MS fragment 17 denoted OH, not NH_3_ gas; similarly, fragment 30 denoted NO, not CH_2_O gas. The disappearance of NH_3_ in the IR spectroscopy of the NC/ADN mixture was assumed to be because the NH_3_ was oxidized by NO_2_ to form N_2_ and H_2_O. Similarly, the aldehyde disappearance may be due to the aldehyde being oxidized by NO_2_ or O_2_ to form CO and CO_2_. As the NC/ADN mixture started to decompose, instead of NO_2_, N_2_O (1274 cm^−1^, 2201 cm^−1^) began to form at 139.3 °C, which was 5.5 °C less than that of N_2_O formation in the decomposition process of ADN. On the contrary, NO_2_ was detected for the NC/ADN mixture until the temperature reached 148.2 °C, which was 7 °C higher than that of NO_2_ release in the decomposition process of ADN, which indicates that NC can accelerate the decomposition of ADN to form N_2_O and inhibit the formation of NO_2_. Compared to decomposition of ADN or NC, it can be seen that the main thermal decomposition process of the NC/ADN mixture can be divided into two stages. In the first stage, ADN decomposition dominates the decomposition process, with N_2_O formation being the main decomposition pathway. Then, oxidative gases such as O_2_ and HNO_2_ appear in the following process, while NO_2_ formation resulting from the N-NO_2_ breakage of ADN or the O-NO_2_ breakage of NC coexist as minor pathways. In the second stage, the intermediate thermal decomposition products of ADN reacted quickly with NC to form gases such as N_2_O, NO, CO and HCN, wherein NH_3_ and aldehyde were oxidized completely to form N_2_, CO_2_ and H_2_O. NC inhibited the dissociation of the N-NO_2_ bond of ADN to form NO_2_ and accelerated the rearrangement of the ADN anion to produce the gas N_2_O in the initial decomposition stage of the NC/ADN mixture.

### 2.7. Interaction Mechanism of NC with ADN

**Figure 7:**
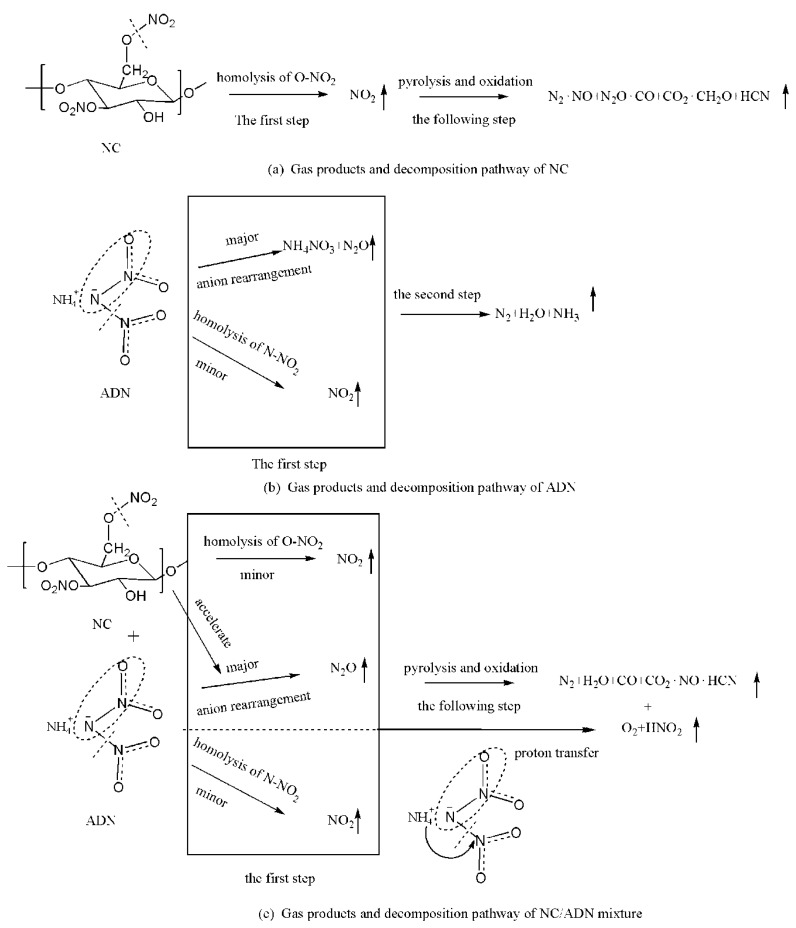
Decomposition pathway of NC, ADN and NC/ADN mixture.

The initial interaction mechanism was proposed preliminarily based on the gas products of the NC/ADN mixture and the decomposition pathways of NC and ADN. The proposed decomposition scheme is illustrated in Figure 7. 

The initial decomposition of NC began with the hemolysis of the O-NO_2_ bond to form NO_2_ gas first, then N_2_O appeared as the secondary product of NO_2_ reacted with the residue of NC. However, N_2_O was the significant product in the first decomposition step of and; hence, the N_2_O formation in the initial stage can be completely ascribed to the decomposition of ADN. It is hard to discriminate the origin of NO_2_ in the initial decomposition stage of the NC/ADN mixture, because both ADN and NC can produce it from the breakup of N-NO_2_ or O-NO_2_, respectively. For ADN alone, no O_2_ and HNO_2_ formed; however, these two oxidative gases formed for the NC/ADN mixture. In the initial decomposition stage of the NC/ADN mixture, NC did not change the major gas products of ADN. However, the acceleration effect of NC on ADN to form N_2_O can be confirmed from the intensity of the N_2_O fragment in the MS test. We speculate that NC can make proton transfer or the combination of protons and anions easier. In this paper, we cannot promise that the protons only came from NH_4_^+^, due to the hydroxide group in the NC molecule also being able to provide protons. Judging from the generation time of O_2_ and HNO_2_, it is sure that proton transfer did not happen in the initial stage of the NC/ADN mixture. Hence, it is certain that NC did not change the initial decomposition pathway of ADN. The disappearance of NH_3_ and CH_2_O can be ascribed to complete oxidation of NH_3_ and CH_2_O by oxidative gases such as O_2_, N_2_O and NO_2_.

## 3. Materials and Methods

### 3.1. Raw Materials

ADN with 99% purity and NC with 11.97 wt% nitrogen content were prepared by Xi’an Modern Chemistry Research Institute. The NC/ADN mixture was made by adding ADN powders in NC acetone solution (10 wt%) with the mass ratio of NC/ADN 1:3 followed by stirring for 10 min. The solution was then poured into a glass dish. A dried membrane composed of NC and ADN was prepared after the mixed solution was kept in a desiccator at room temperature for 24 h.

### 3.2. Thermal Behavior in Open Circumstances

The DSC-TG curves were obtained from DSC-TG-FTIR-MS experiments in an open aluminum crucible with inert argon (Ar) gas flow of 50 mL/min and a heating rate of 10 °C/min. The masse of ADN, NC, and the NC/ADN mixture were about 1.5 mg.

### 3.3. Thermal Behavior in Closed Circumstances

The DSC experiments of ADN, NC and the NC/ADN mixture were performed using a calorimeter (Model Q200, TA company, Newcastle, NSW, Austrial) via hermetic gold-plated crucibles with volume 27 μL and sample mass 0.8 mg at the heating rates of 1 °C/min, 5 °C/min, 10 °C/min, and 20 °C/min.

### 3.4. Thermal Behaviors in Quasi-Adiabatic Circumstances

The ARC tests were investigated using a calorimeter (Model ARC 254, NETZSCH company) with the detection threshold of 0.02 °C/min, temperature step of 5 °C and heating rate of 5 °C/min. The masses of NC, ADN and NC/ADN for the ARC tests were 100 mg.

### 3.5. Thermal Behavior under Constant Temperature Conditions

**Figure 8:**
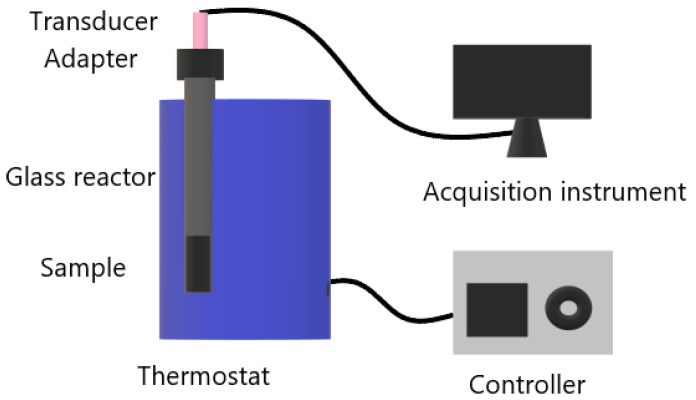
The diagrammatic sketch of the instrument for measuring gas pressure.

The thermal decomposition gas pressures of ADN, NC and the NC/ADN (mass ratio is 1/3) mixture under constant temperature were measured using a self-developed instrument with the diagrammatic sketch shown in Figure 8. The measurement accuracy of the pressure transducer was ±0.1%. The temperature was kept constant at 100 ± 0.2 °C.

#### 3.5.1. Vacuum Tests

The thermostat was preheated to 100 °C. Sample with a mass of 28 mg was loaded into the glass reactor. The glass reactor was vacuumed until the pressure fell below 100 Pa and sealed. The sealed glass reactor was put into the thermostat, and the gas pressure in the glass reactor was recorded simultaneously. The test time was 40 h.

#### 3.5.2. Air Atmosphere Tests

The sealed glass reactor with 28 mg of sample was directly put into the thermostat without vacuuming, and other conditions were identical with those of the vacuum tests.

### 3.6. Gas Products Measurement

The thermal decomposition gas products of ADN, NC and the NC/ADN mixture were respectively detected using MS and FTIR in DSC-TG-FTIR-MS experiments with a heating rate of 10 °C/min and a mass of 1.5 mg. The experiments were carried out on a combined set composed of a STA 449 C DSC-TG module (NETASCH Company, Selbu, Germany), MS QMS 403 C (NETASCH Company, Selbu, Germany), and the infrared spectrometer Nicolet 5700 FTIR (Thermo Fisher Company, Madison, WI, USA).

## 4. Conclusions 

In the initial decomposition stage of the NC/ADN mixture, the decomposition of ADN was predominant; in particular, the formed gas N_2_O was the critical factor to affect the compatibility of NC with ADN, which can be substantiated by the successful inhibition of further decomposition of the NC/ADN mixture under high vacuum conditions. The addition of NC into ADN reduced the initial thermal decomposition temperature of ADN significantly by promoting N_2_O formation through the rearrangement of ADN anion. Consequently, the preference to form N_2_O over NO_2_ resulted in the generation of new oxidative gases such as O_2_ and HNO_2_. Subsequently, the generated nitrogen oxide (NO_X_) and HNO_2_ initiated the decomposition of NC, followed by the intensive interaction of NC residue with O_2_ accompanied by a large amount of heat release. However, the reason why NC can accelerate the rearrangement of ADN anion to produce N_2_O is unknown. Without question, this is the next project worthy of exploration to resolve the chemical compatibility of NC with ADN.

## Figures and Tables

**Table 1:** The peak temperature and *E*_a_ of ADN, NC and NC/ADN mixture.

Sample	Peak Temperature (°C)				*E*_a_/(kJ·mol^−1^)
	1 °C/min	2 °C/min	5 °C/min	10 °C/min	
ADN	155.3	168.8	180.8	199.3 (185.8)	83.2 (R^2^ = 0.98)
NC	188.4	195.6	208.3	216.3 (208.0)	143.7 (R^2^ = 0.99)
NC/ADN mixture	155.0	161.9	181.1	188.6 (172.0)	96.8 (R^2^ = 0.98)

Note: The temperature value in brackets denotes the peak temperature of material obtained from DSC-TG tests in open aluminum crucibles at heating rate of 10 °C/min with argon gas flow rate of 50 mL/min. The *R*^2^ value in brackets denotes the coefficient of determination.

**Table 2:** The characteristic temperature and time of ADN, NC, NC/ADN mixture under quasi-adiabatic conditions.

Sample	Temperature at the INITIAL Detection Point (°C)	Heating Rate at the Initial Detection Point (°C/min)	Heating Time at the Initial Detection Point (min)	The Maximum Temperature Rise Rate (°C/min)	Time to the Maximum Temperature Rise Rate (min)
ADN	126.1	0.14	843.3	44.5	48.2
NC	152.6	0.03	1076.9	113.6	87.5
NC/ADN mixture	106.4	0.70	585.5	107.2	9.6

**Table 3:** Characteristic pressure data of ADN, NC and NC/ADN mixture.

Sample	Initial Condition (Vacuum)			Initial Condition (Air Atmosphere)		
	Initial Pressure (kPa)	Pressure after 40 h (kPa)	Pressure Difference (kPa)	Initial Pressure (kPa)	Pressure after 40 h (kPa)	Pressure Difference (kPa)
ADN	1.01	1.96	0.95	116.71	119.62	2.91
NC	0.58	0.74	0.16	118.65	118.78	0.13
NC/ADN mixture	1.15	4.54	3.39	120.15	247.31	127.16

**Table 4:** Mass fragments and assignments of the thermal decomposition products of ADN.

Charge Mass Ratio	Assignment	Initial Temperature (°C)	Peak Temperature (°C)	Relatively Intensity × 10^11^ (%)
14	N	152.0	191.4	2.48
15	NH	152.4	191.4	0.15
16	O, NH_2_	157.4	192.4	2.40
17	OH, NH_3_	153.4	202.4	4.00
18	H_2_O	157.9	202.9	21.4
28	N_2_	147.9	192.4	21.1
30	NO	143.9	187.4	2.74
32	O_2_	None	None	None
44	N_2_O	146.9	187.4	7.22
45	HNNO	155.4	188.4	0.11
46	NO_2_	147.9	196.9	0.07
47	HNO_2_	None	None	None
63	HNO_3_	None	None	None

**Table 5:** Fragments and assignments of the thermal decomposition products of NC.

Charge Mass Ratio	Assignment	Initial Temperature (°C)	Peak Temperature (°C)	Relatively Intensity × 10^11^ (%)
16	O	185.2	210.6	3.12
17	OH	195.4	214.2	3.53
18	H_2_O	190.3	214.9	19.3
26	CN	190.4	209.1	0.16
27	HCN	194.9	212.4	0.65
28	N_2_, CO	185.9	210.8	26.5
29	CHO	188.9	211.4	0.66
30	NO, CH_2_O	170.5	208.9	4.87
32	O_2_	None	None	None
43	NCO	191.9	211.4	0.13
44	N_2_O, CO_2_	178.9	210.9	6.35
46	NO_2_	170.5	205.9	0.09
47	HNO_2_	None	None	None
63	HNO_3_	None	None	None

**Table 6:** Fragments and assignments of the thermal decomposition products of NC/ADN mixture.

Charge Mass Ratio	Assignment	Initial Temperature (°C)	Peak Temperature (°C)	Relatively Intensity × 10^11^ (%)
16	O	156.1	171.5	34.6
17	OH	155.3	171.8	34.5
18	H_2_O	158.8	175.8	61.0
26	CN	156.8	171.8	1.11
27	HCN	160.8	176.3	1.05
28	N_2_, CO	152.8	176.3	56.0
29	CHO	159.8	176.3	1.11
30	NO	145.3	176.3	19.3
32	O_2_	162.8	175.7	7.69
43	NCO	146.3	175.8	0.23
44	N_2_O, CO_2_	147.8	171.8	239
46	NO_2_	144.8	171.8	1.77
47	HNO_2_	164.3	171.8	0.07
63	HNO_3_	None	None	None

**Table 7:** Gas products and assignments of the thermal decomposition products of ADN.

Wavelength (cm^−1^)	Assignment	Initial Temperature (°C)			Peak Temperature (°C)		
		ADN	NC	NC/ADN Mixture	ADN	NC	NC/ADN Mixture
889	NH_3_	177.6	——	——	201.1	——	——
1274	N_2_O	144.8	199.0	139.3	190.5	211.7	172.9
1309	N_2_O	152.1	——	146.4	195.5	——	169.3
1597	NO_2_	146.7	177.3	148.2	206.7	202.7	174.6
1631–1635	NO_2_	141.2	166.5	153.5	206.7	202.7	169.4
1706	NH_3_	181.2	——	——	199.4	——	——
1720	CHO, HCOOH	——	184.6	——	——	208.1	——
1745	CHO, HCOOH	——	199.0	——	——	211.7	——
1911	NO	——	186.4	169.3	——	206.3	172.9
2113–2119	CO	——	189.9	169.3	——	209.9	172.9
2201–2205	N_2_O	148.5	195.4	139.3	190.3	209.9	172.9
2238	N_2_O	146.7	197.2	141.1	190.3	211.7	169.3
2345–2353	CO_2_	——	200.8	144.6	——	209.9	169.4

## Data Availability

The data are available on request.

## References

[1] Comtois E., Dubois C., Favis B.D. (2022). Linear burning rate of double base propellant containing Azidodeoxycellulose. J. Energ. Mater..

[2] Touidjine S., Boulkadid K.M., Trache D., Belkhiri S., Mezroua A. (2022). Preparation and Characterization of Polyurethane/Nitrocellulose Blends as Binder for Composite Solid Propellants. Propellants Explos. Pyrotech..

[3] Rodrigo L.B.R., Pedro A.G.B., Nami L.N., Fernando C.P., Maurício F.L., Tanos C.C.F., Letivan G.M.F. (2022). Can green nitrocellulose-based propellants be made through the replacement of diphenylamine by the natural product curcumin?. J. Energ. Mater..

[4] Courty L., Gillard P., Ehrhardt J., Baschung B. (2021). Experimental determination of ignition and combustion characteristics of insensitive gun propellants based on RDX and nitrocellulose. Combust. Flame.

[5] Okada K., Saito Y., Akiyoshi M., Endo T., Matsunaga T. (2021). Preparation and Characterization of Nitrocellulose Nanofiber. Propellants Explos. Pyrotech..

[6] Zhang X., Weeks B.L. (2014). Preparation of sub-micron nitrocellulose particles for improved combustion behavior. J. Hazard. Mater..

[7] Dobrynin O.S., Zharkov M.N., Kuchurov I.V., Fomenkov I.V., Zlotin S.G., Monogarov K.A., Meerov D.B., Pivkina A.N., Muravyev N.V. (2019). Supercritical Antisolvent Processing of Nitrocellulose: Downscaling to Nanosize, Reducing Friction Sensitivity and Introducing Burning Rate Catalyst. Nanomaterials.

[8] Chen F.-Y., Xuan C.-L., Lu Q.-Q., Xiao L., Yang J.-Q., Hu Y.-B., Zhang G.-P., Wang Y.-L., Zhao F.-Q., Hao G.-Z. (2022). A review on the high energy oxidizer ammonium dinitramide: Its synthesis, thermal decomposition, hygroscopicity, and application in energetic materials. Def. Technol..

[9] Pang W.Q., Xu H.X., Li Y., Shi X.B. (2011). Characteristics of NEPE propellant with ammonium dinitramide (ADN). Adv. Mater. Res..

[10] Pang W., Fan X., Yi J., Zhao F., Xu H., Li J., Wang B., Li Y. (2010). Thermal Behavior and Non-isothermal Decomposition Reaction Kinetics of NEPE Propellant with Ammonium Dinitramide. Chin. J. Chem..

[11] Wang Q., Xu L.-P., Deng C.-Q., Yao E.-G., Chang H., Pang W.-Q. (2023). Characterization of Electrospinning Prepared Nitrocellulose (NC)-Ammonium Dinitramide (ADN)-Based Composite Fibers. Nanomaterials.

[12] Zhang L.Y., Heng S.Y., Liu Z.R., Zhang G., Zhao F.Q., Tan H.M. (2009). Interaction of NC/NG with ADN. Chin. J. Energ. Mater..

[13] He S.R., Zhang L.J., Heng S.R., Liu Z.R. (2008). Study on interaction of ADN and (NC+NG) by gasometric method. Chin. J. Energetic Mater..

[14] Li J.Z., Wang W., Liu F.L., Fu X.L., Fan X.Z., Zhang L.Y., Wang Q. (2011). Influences of stabilizers on the nascent interaction between ADN and NC. Chin. J. Explos. Propellants.

[15] Yue P., Heng S.Y., Han F., Zhang L.Y., He S.R. (2008). Compatibility of ADN with five kinds of binders. Chin. J. Energetic Mater..

[16] Dauerman L., Tajima Y.A. (1968). Thermal decomposition and combustion of nitrocellulose. AIAA J..

[17] Makashir P.S., Mahajan R.R., Agrawal J.P. (1995). Studies on kinetics and mechanism of initial thermal decomposition of nitrocellulose Isothermal and non-isothermal techniques. J. Therm. Anal. Calorim..

[18] Wang Y., Liu R., Ning B.K., Pan Q., Hu R.Z. (1998). A study of the thermal decomposition mechanism of of nitrocellulose. Chin. J. Energ. Mater..

[19] Chai H., Duan Q., Cao H., Li M., Sun J. (2020). Effects of nitrogen content on pyrolysis behavior of nitrocellulose. Fuel.

[20] Brill T.B., Gongwer P.E. (1997). Thermal Decomposition of Energetic Materials 69. Analysis of the kinetics of nitrocellulose at 50 °C–500 °C. Propellants Explos. Pyrotech..

[21] Anatolii I.K., Yurii I.R., Georgii B.M. (1999). Kinetics and Mechanism of Thermal Decomposition of Dinitramide. Propell Explos. Pyrot..

[22] Ermolin N.E., Fomin V.M. (2016). On the mechanism of thermal decomposition of ammonium dinitramide (review). Combust. Explos. Shock. Waves.

[23] Yang R., Thakre P., Yang V. (2005). Thermal Decomposition and Combustion of Ammonium Dinitramide (Review). Combust. Explos. Shock. Waves.

[24] Kumar P. (2018). An overview on properties, thermal decomposition, and combustion behavior of ADN and ADN based solid propellants. Def. Technol..

[25] Wang K., Xue B., Chen J.-G., He Z.-H., Ji Y., Wang B., Lu J., Liu Z.-W., Liu Z.-T. (2020). A combined experimental and theoretical study of the thermal decomposition mechanism and kinetics of ammonium dinitramide (ADN). New J. Chem..

[26] Oxley J.C., Smith J.L., Zheng W., Rogers E., Coburn M.D. (1997). Thermal Decomposition Studies on Ammonium Dinitramide (ADN) and ^15^N and ^2^H Isotopomers. J. Phys. Chem. A.

[27] Izato Y.-I., Koshi M., Miyake A., Habu H. (2017). Kinetics analysis of thermal decomposition of ammonium dinitramide (ADN). J. Therm. Anal. Calorim..

[28] Babrauskas V., Leggett D. (2020). Thermal decomposition of ammonium nitrate. Fire Mater..

[29] Izato Y.-I., Miyake A. (2015). Thermal decomposition of molten ammonium nitrate (AN). J. Therm. Anal. Calorim..

[30] Pavlov A.N., Grebennikov V.N., Nazina L.D., Nazin G.M., Manelis G.B. (1999). Thermal decomposition of ammonium dinitramide and mechanism of anomalous decay of dinitramide salts. Russ. Chem. Bull..

[31] Hu R.Z., Gao S.L., Zhao F.Q., Shi Q.Z., Zhang T.L., Zhang J.J. (2008). Thermal Analysis Dynamics.

[32] Jin M., Luo N., Li G., Luo Y. (2015). The thermal decomposition mechanism of nitrocellulose aerogel. J. Therm. Anal. Calorim..

